# Tooth loss and associated self-rated health and psychological and subjective wellbeing among community-dwelling older adults: A cross-sectional study in India

**DOI:** 10.1186/s12889-021-12457-2

**Published:** 2022-01-04

**Authors:** T. Muhammad, Shobhit Srivastava

**Affiliations:** International Institute for Population Sciences, Mumbai, Maharashtra 400088 India

**Keywords:** Tooth loss, Self-assessed health, Older adults, India

## Abstract

**Background:**

Losing teeth has been considered as part of normal aging. However, in developing countries, tooth loss among older adults is shown to be more negatively associated with self-image and quality of life compared to their western counterparts. This study examines the association of tooth loss with self-rated health and psychological and subjective wellbeing among older adults in India.

**Methods:**

Data were derived from the "Building Knowledge Base on Population Ageing in India" (BKPAI) survey which was carried out in 2011. The final sample size for the analysis was 9231 older adults. Descriptive statistics and bivariate analysis along with binary logistic regression analysis were conducted to fulfil the objective of the study.

**Results:**

A proportion of 12.3% of older adults reported complete tooth loss. It was found that older adults who reported tooth loss were 2.38 times significantly more likely to have poor self-rated health (SRH) [2.38; CI: 1.99,2.83] than older adults who did not report tooth loss. The odds of low psychological health were high among older adults who suffered from tooth loss than their counterparts [OR: 1.59; CI: 1.33,1.91]. Older adults who reported tooth loss had 65% significantly higher odds of low subjective well-being than older adults who did not report tooth loss [OR: 1.65; CI: 1.38,1.97].

**Conclusion:**

Complete loss of teeth is associated with older individuals’ poor SRH as well as low psychological and subjective well-being, but such a consequence is avoidable by practising the efforts to maintain good oral health.

## Background

Losing teeth progressively has been considered as part of normal aging [1]. However, in developing countries, tooth loss among older adults is shown to be more negatively associated with self-image and quality of life compared to their western counterparts [2, 3]. Study reported that quite a few study participants discussed tooth loss with their family and friends showing the society’s perception of tooth loss as a social stigma despite the acceptance of tooth loss as normal aging procedure in western countries [2].

Findings of a study with 85-year-old participants indicated that older adults with more than 20 teeth had better subjective physical health than those who had less than 19 teeth [4]. A recent large population-based survey indicates that even loss of a few teeth may indicate an increased risk of cardiovascular diseases, diabetes, or all-cause mortality among older adults [5]. Furthermore, edentulism or complete loss of teeth significantly affects health related quality of life in older adults and the use of dental restorations do not improve their perceived quality of life [6]. Results from a review of 35 studies suggest that tooth loss is associated with unfavourable oral health-related quality of life (OHQoL) among older adults [7]. Significant associations between measures of oral-health problems, pain, and disability and three broader measures of psychological well-being were also revealed. Older adults in a study who rated their oral health as poor had poor self-rated general health than their counterparts who rated their oral health favourably [8].

Previous studies suggest that loss of teeth can also affect older people’s ability to interrelate with others and can have a considerable impact on their lifestyle due to problems with communication in family and social interactions, causing isolation and depression [9–11]. A growing body of literature shows that physical fitness, psychological well-being and health behaviours such as exercise, alcohol consumption, smoking and obesity are associated with self-rated health (SRH) [12, 13]. Participation in exercise, balanced diet and non-smoking over the time predicted good SRH among older adults at follow-up [14]. Moreover, general health and oral health among older population were associated, although oral health impact was only associated with general health for those with more health problems, indicating that those in worse health suffer more problems from tooth loss [8, 15].

The loss of teeth that results in low self-esteem and poor performance of the individual in society proved to have a significant association with social participation [16]. Previous research has documented the effect of complete tooth loss or edentulism on facial appearance, speech, and eating that may have an impact on mental health and well-being through consequences such as low self-esteem, and decline in social activities due to embarrassment [16, 17]. Studies have also examined the socio-demographic and health determinants of tooth loss and edentulism among older population in India [18–21]. On the other hand, the challenges to accessing dental care services including awareness, financial barriers, traditional beliefs, trust and convenience were well documented in prior research [22, 23].

Since dental care in India as in any other developing countries is considered entirely as a private matter at the expense of patients depending on their subjective considerations, few data is available and the studies on association of tooth loss with the health outcomes of older adults are scant. Since dentition status provides information pertinent to older adults’ general health, we in this study examine the association of tooth loss with the subjective assessment of health outcomes among older adults in India. The study hypothesised that there is a significant association of tooth loss with subjective assessment of health among older adults in India.

## Methods

### Data

Data for this study are derived from the BKPAI survey (Building Knowledge Base on Population Ageing in India). The survey was carried out in India in 2011 [24]. A primary survey was conducted in seven states of India (Himachal Pradesh, Punjab, West Bengal, Odisha, Maharashtra, Kerala and Tamil Nadu), that covered a total of 9852 older adults from 8329 households in rural and urban areas [24]. The surveyed states have a higher percentage of the 60+ population compared to the national average and these states also represent all regions of the country [24]. The individual questionnaire was used which covers the socio-demographic profile, work history, and benefit, income, and assets, living arrangement, social activities, the health status of older adults and social security related question [24]. The BKPAI sample design entails a two-stage probability sampling, where first, villages were classified into different strata on the basis of population size, and the number of PSUs (Primary sampling units) to be selected was determined in proportion to the population size of each stratum. Using probability proportional to population size (PPS) technique, the PSUs have been chosen, and within each selected PSU, households of older adults were selected through systematic sampling. A similar procedure was applied in drawing samples from urban areas [24]. Because this was a study of the elderly, the sample size was split evenly across urban and rural locations, regardless of the proportion of the population in each. The study included 80 PSUs (villages or urban wards) – 40 urban and an equal number of rural – with 16 elderly households per PSU. The questionnaires for each state were bilingual, with questions in both the primary language of the states and English. The final sample size for the analysis was 9231 older adults. The study protocol is performed in accordance with the relevant guidelines. The ethical approval for the study was obtained from the university of Southampton and written informed consent was obtained from the human participants in the study.

### Variable description

#### Outcome variables

In India, study found that SRH has a satisfactory construct validity which is even stronger than that in an advanced country showing its association with different health dimensions [25]. SRH in the current study have a scale of 1 to 5, “excellent, very good, good, fair and poor” and was categorized as 0 “good” (representing good, very good and excellent) and 1 “poor” (representing poor or fair) [26]. The 12-item version of the General Health Questionnaire (GHQ-12) was used as a measure of low psychological health. The GHQ-12 is an influential and reliable self-reported screening tool commonly used for identifying the non-specific and minor psychiatric disorder in the general population [27, 28]. Psychological health was having a scale of 0 to 12 on the basis of experiencing stressful symptoms and was recoded as 0 “high” (representing 6+ scores) and 1 “low” (representing score 5 and less) [29, 30]. The low psychological health represents lower levels of psychological health or psychological distress among older adults (Cronbach's alpha: 0.90) [31].

The 9-item subjective well-being (SWB) questionnaire was used to measure low subjective well-being. Subjective well-being have a scale of 0 to 9 and was categorized as 0 “high” experiencing better experience (representing 6+ scores) and 1 “low” experiencing negative experience (representing score 5 and less) [32]. Twelve questions on psychological health and nine questions on subjective well-being were asked to assess the outcome variables. All the questions were asked on Likert scales and were recoded and used accordingly as per literature [26]. The low subjective well-being represents lower levels of subjective well-being among older adults (Cronbach alpha: 0.93) [12].

#### Explanatory variables

The main explanatory variable for the current study is complete loss of teeth (permanent). The variable was assessed using the question asked under the chronic morbidities regarding the ‘loss of all natural teeth’ which was categorized as no and yes.

Smoking tobacco, chewing tobacco and Alcohol consumption was categorized as no and yes. Ability to do activities of daily living (ADL) was having a scale of 0 to 6 where in it represents higher the score higher the independence. A score of was categorized as 0 “no” which represents full independence and 5 and less was categorized as 1 “yes” which represents not fully independent to do activities of daily living (Cronbach Alpha: 0.93) [12]. Ability to do instrumental activities of daily living (IADL) was having a scale of 0 to 8 representing higher the score higher the independence. A score of 6+ was categorized as 0 “no” representing high IADL and score of 5 and less was recoded as 1 “yes” representing low IADL [12, 33].

Age was categorized as 60-69, 70-79 and 80 + years. Sex was categorized as male and female. Marital status was categorized as not in union “included never married, widowed, divorced and separated” and currently in union. Educational status was categorized as no schooling, below 5 years of schooling, 6-10 years of schooling and 11 and above years of schooling. Working status was categorized as no and yes. The reference period was last 12 months. Living arrangement was categorized as living alone, with spouse, with children and other. Wealth quintile was computed using 30 household assets and was divided into 5 quintiles as poorest, poorer, middle, richer, richest. Religion was categorized as Hindu, Muslim, Sikh and others. Caste was categorized as Scheduled Caste, Scheduled Tribes, Other Backward Class and others. Place of Residence was categorized as rural and urban. Data for seven states was available in the data as mentioned in the data section.

### Statistical analysis

Descriptive statistics and bivariate analysis were used to find the preliminary results. Further, binary logistic regression analysis [34] was used to fulfil the objective of the study. The outcome variable was SRH coded as 0 “good” and 1 “poor”, low psychological health recoded as 0 “high” and 1 “low” and subjective well-being recoded as 0 “high” and 1 “low”. The results were presented in the form of adjusted odds ratio (AOR) with a 5% significance level.

The model [34] is usually put into a more compact form as follows:

$$\ln \left(\frac{P_i}{1-{P}_i}\right)={\beta}_0+{\beta}_1{x}_1+\dots +{\beta}_M{x}_{m-1},$$

Where *β*_0_, …. . , *β*_*M*_ are regression coefficients indicating the relative effect of a particular explanatory variable on the outcomes. These coefficients change as per the context in the analysis in the study [34]. Variance inflation factor (VIF) [35] was calculated to measure multi-collinearity among the variables and it was revealed from the results that there was no evidence of multi-collinearity among the variables. Additionally, svyset command was used to control the analysis for complex survey design and individual weight present in the dataset was used to make the estimates representative.

## Results

Socio-economic and demographic profile of older adults were described in Table 1. About 12.3% of older adults suffer from tooth loss. Nearly 15, 22 and 8% of older adult’s smoke tobacco, chew tobacco and consume alcohol. About 64% of older adults suffer from one or more chronic diseases. Almost 8% and 57% of older adults suffers from ADL and IADL. Nearly one-tenth of older adults were from age group 80 years and above. About 53% of older adults are female and 47% are male. Almost 61% of the older population was currently in union. Surprisingly, half of the older adults had no education at all whereas 6% had 11 and more years of education. About 9% of older adults were retired at the time of survey. Nearly 6% of the older adults were living alone and 16% were living with their spouse. About one-fourth of the older adults belong to poorest wealth status. Nearly 8 in 10 older adults were from Hindu religion and 2 in 10 from Scheduled Caste category. About one-fourth of the older adults live in urban areas.

**Table 1:** Socio-economic and demographic profile of older adults

Background factors	Sample	Percentage
**Tooth loss**		
No	8095	87.7
Yes	1136	12.3
**Smoking tobacco**		
No	7809	84.6
Yes	1422	15.4
**Chewing tobacco**		
No	7228	78.3
Yes	2003	21.7
**Alcohol consumption**		
No	8525	92.4
Yes	706	7.7
**Chronic diseases**		
No	3268	35.4
Yes	5963	64.6
**Difficulty in ADL**		
High	8541	92.5
Low	690	7.5
**Difficulty in IADL**		
High	4008	43.4
Low	5223	56.6
**Age (years)**		
60-69	5704	61.8
70-79	2536	27.5
80+	991	10.7
**Sex**		
Male	4372	47.4
Female	4859	52.6
**Marital Status**		
Not in union	3649	39.5
Currently in union	5582	60.5
**Education**		
No education	4684	50.7
Below 5 years	1900	20.6
6-10 years	2086	22.6
11+ years	562	6.1
**Working status (last one year)**		
No	6212	67.3
Yes	2223	24.1
Retired	796	8.6
**Living arrangement**		
Alone	547	5.9
With spouse	1467	15.9
With children	6493	70.3
Others	725	7.9
**Wealth quintile**		
Poorest	2169	23.5
Poorer	2029	22.0
Middle	1913	20.7
Richer	1720	18.6
Richest	1399	15.2
**Religion**		
Hindu	7324	79.3
Muslim	651	7.1
Sikh	870	9.4
Others	386	4.2
**Caste**		
Scheduled Caste	1911	20.7
Scheduled Tribe	515	5.6
Other Backward Class	3364	36.4
Others	3441	37.3
**Residence**		
Rural	6827	74.0
Urban	2404	26.0
**State**		
Himachal Pradesh	1471	15.9
Punjab	1279	13.9
West Bengal	1128	12.2
Orissa	1454	15.8
Maharashtra	1229	13.3
Kerala	1341	14.5
Tamil Nadu	1330	14.4
**Total**	9231	100.0

*ADL* Activities of Daily Living, *IADL* Instrumental Activities of Daily Living

**Fig. 1:**
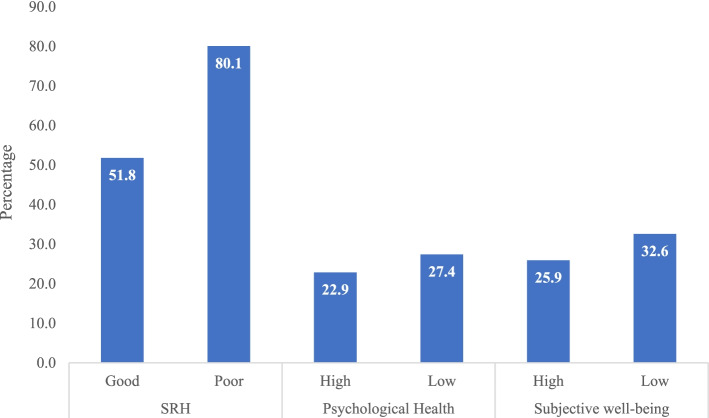
Percentage of older adults suffering from tooth loss with poor SRH, low psychological health and low subjective well-being

Figure 1 depicts percentage of tooth loss among older adults with poor SRH, low psychological health and low subjective well-being. The prevalence of poor SRH (80.1%), low psychological health (27.4%) and low subjective well-being (32.6%) was high among older adults who suffered from tooth loss.

Table 2 represents percentage of older adults reporting poor SRH, low psychological health and low subjective well-being by background characteristic. Prevalence of poor SRH was high among older adults who smoke tobacco (60%), chew tobacco (57%), had chronic disease (63%) and with low ADL (85%) and IADL (63%). Prevalence of low psychological health was high among older adults who chew tobacco (30%), had chronic disease (25%) and with low ADL (51%) and IADL (30%). Prevalence of low subjective well-being was high among older adults who chew tobacco (34%), had chronic disease (29%) and with low ADL (55%) and IADL (35%). Apart from that poor SRH, low psychological health and low subjective well-being was significantly associated with age, gender, marital status, educational status, working status, living arrangement, wealth status, religion, caste and residential status.

**Table 2:** Percentage of older adults reporting poor SRH, low psychological health and low subjective well-being

Background factors	Poor SRH		Low psychological health		Low subjective well-being	
	%	***p*** < 0.05	%	***p*** < 0.05	%	***p*** < 0.05
**Smoking tobacco**		0.046		0.936		0.455
No	54.8		23.7		26.9	
Yes	57.9		22.1		25.7	
**Chewing tobacco**		0.032		< 0.001		< 0.001
No	54.9		21.5		24.8	
Yes	56.8		30.3		33.7	
**Alcohol consumption**		0.093		0.031		< 0.001
No	55.5		23.7		27.4	
Yes	53.1		19.8		18.6	
**Chronic diseases**		< 0.001		< 0.001		< 0.001
No	40.5		20.0		23.3	
Yes	63.4		25.3		28.6	
**Difficulty in ADL**		< 0.001		< 0.001		< 0.001
High	52.9		21.2		24.5	
Low	84.7		51.3		54.8	
**Difficulty in IADL**		< 0.001		< 0.001		< 0.001
High	45.5		14.4		16.1	
Low	62.8		30.4		34.9	
**Age (years)**		< 0.001		< 0.001		< 0.001
60-69	49.6		19.9		23.2	
70-79	62.2		27.0		30.0	
80+	70.0		34.9		38.7	
**Sex**		< 0.001		< 0.001		0.048
Male	51.9		21.1		23.9	
Female	58.3		25.5		29.3	
**Marital Status**		< 0.001		< 0.001		< 0.001
Not in union	61.3		28.5		32.9	
Currently in union	51.3		20.1		22.7	
**Education**		< 0.001		< 0.001		< 0.001
No education	61.0		30.6		35.5	
Below 5 years	56.9		22.3		24.0	
6-10 years	44.8		12.5		13.9	
11+ years	41.1		8.4		10.3	
**Working status (last one year)**		< 0.001		< 0.001		< 0.001
No	60.7		27.1		30.4	
Yes	47.1		19.4		23.5	
Retired	35.8		6.2		7.0	
**Living arrangement**		< 0.001		< 0.001		< 0.001
Alone	56.7		32.9		39.7	
With spouse	49.9		20.7		25.8	
With children	55.8		22.9		25.4	
Others	60.7		26.3		30.9	
**Wealth quintile**		< 0.001		< 0.001		< 0.001
Poorest	61.8		37.1		47.3	
Poorer	56.0		29.6		32.4	
Middle	53.2		19.7		21.1	
Richer	48.5		14.7		14.7	
Richest	55.2		9.2		9.3	
**Religion**		< 0.001		< 0.001		< 0.001
Hindu	52.8		25.7		28.5	
Muslim	66.3		23.1		29.9	
Sikh	65.5		7.8		12.1	
Others	61.4		16.3		21.6	
**Caste**		< 0.001		< 0.001		< 0.001
Scheduled Caste	60.0		28.0		33.8	
Scheduled Tribe	46.5		32.4		35.1	
Other Backward Class	53.2		25.7		27.8	
Others	56.0		17.3		20.5	
**Residence**		< 0.001		< 0.001		< 0.001
Rural	56.9		25.1		28.3	
Urban	50.8		18.7		22.4	
**State**		< 0.001		< 0.001		< 0.001
Himachal Pradesh	46.0		17.0		15.1	
Punjab	65.9		7.2		11.4	
West Bengal	77.9		29.3		48.3	
Orissa	47.5		37.5		35.3	
Maharashtra	40.9		22.7		34.3	
Kerala	67.1		13.8		14.8	
Tamil Nadu	46.1		36.2		31.7	

*if *p* < 0.05, *%* percentage, *SRH* self-rated health, *ADL* Activities of Daily Living, *IADL* Instrumental Activities of Daily Living, *p*-value based on chi-square test

Table 3 reveals logistic regression estimates for poor SRH, low psychological health and low subjective well-being among older adults. The regression estimates were adjusted for other potential factors that affect the outcome variables as well. It was found that older adults who suffered from tooth loss were 2.38 times significantly more likely to report poor SRH [OR: 2.38; CI: 1.99,2.83] than the older adults who did not suffered from tooth loss. The odds for low psychological health was high among older adults who suffered from tooth loss than their counterparts [OR: 1.59; CI: 1.33,1.91]. Older adults who suffers from tooth loss had 65% significantly higher likelihood to suffer from low subjective well-being than older adults who did not suffered from tooth loss [OR: 1.65; CI: 1.38,1.97].

**Table 3:** Logistic regression estimates for poor SRH, low psychological health and low subjective well-being among older adults

Background factors	Poor SRH	Low psychological health	Low subjective well-being
	AOR (95% CI)	AOR (95% CI)	AOR (95% CI)
**Tooth loss**			
No	Ref.	Ref.	Ref.
Yes	2.38*(1.99,2.83)	1.59*(1.33,1.91)	1.65*(1.38,1.97)
**Smoking tobacco**			
No	Ref.	Ref.	Ref.
Yes	1.04 (0.88,1.21)	1.11 (0.92,1.34)	1.08 (0.89,1.39)
**Chewing tobacco**			
No	Ref.	Ref.	Ref.
Yes	1.04 (0.92,1.18)	0.99 (0.86,1.13)	0.87*(0.76,0.99)
**Alcohol consumption**			
No	Ref.	Ref.	Ref.
Yes	0.88 (0.72,1.07)	1.14 (0.89,1.45)	0.78 (0.61,1)
**Chronic diseases**			
No	Ref.	Ref.	Ref.
Yes	2.17*(1.91,2.34)	1.57*(1.39,1.79)	1.40*(1.31,1.67)
**Difficulty in ADL**			
High	Ref.	Ref.	Ref.
Low	2.44*(1.94,3.06)	2.27*(1.88,2.74)	2.61*(2.15,3.14)
**Difficulty in IADL**			
High	Ref.	Ref.	Ref.
Low	1.63*(1.47,1.81)	1.68*(1.47,1.91)	1.72*(1.52,1.94)
**Age (years)**			
60-69	Ref.	Ref.	Ref.
70-79	1.18*(1.06,1.32)	1.14 (1,1.3)	1.19*(1.05,1.35)
80+	1.18 (0.99,1.41)	1.19 (0.98,1.43)	1.26*(1.04,1.51)
**Sex**			
Male	Ref.	Ref.	Ref.
Female	0.89 (0.79,1.01)	0.88 (0.76,1.02)	0.94 (0.78,1.04)
**Marital Status**			
Not in union	Ref.	Ref.	Ref.
Currently in union	0.99 (0.88,1.12)	0.97 (0.84,1.12)	0.91 (0.79,1.04)
**Education**			
No education	Ref.	Ref.	Ref.
Below 5 years	0.89 (0.78,1.01)	0.75*(0.65,0.87)	0.74*(0.64,0.86)
6-10 years	0.69*(0.6,0.79)	0.48*(0.4,0.57)	0.53*(0.45,0.63)
11+ years	0.60*(0.48,0.75)	0.51*(0.36,0.71)	0.51*(0.37,0.7)
**Working status (last one year)**			
No	Ref.	Ref.	Ref.
Yes	0.77*(0.68,0.88)	0.81*(0.7,0.94)	0.83*(0.71,0.95)
Retired	0.63*(0.53,0.75)	0.46*(0.34,0.62)	0.47*(0.35,0.62)
**Living arrangement**			
Alone	Ref.	Ref.	Ref.
With spouse	0.99 (0.78,1.26)	0.61*(0.46,0.79)	0.82 (0.61,1.04)
With children	1.01 (0.82,1.25)	0.73*(0.58,0.92)	0.71*(0.57,0.89)
Others	0.93 (0.72,1.21)	0.93 (0.69,1.24)	0.89 (0.67,1.18)
**Wealth quintile**			
Poorest	Ref.	Ref.	Ref.
Poorer	0.67*(0.58,0.79)	0.94 (0.8,1.1)	0.69*(0.59,0.81)
Middle	0.55*(0.46,0.65)	0.76*(0.63,0.92)	0.48*(0.4,0.58)
Richer	0.49*(0.41,0.59)	0.65*(0.52,0.8)	0.41*(0.34,0.51)
Richest	0.52*(0.42,0.64)	0.51*(0.4,0.66)	0.28*(0.22,0.35)
**Religion**			
Hindu	Ref.	Ref.	Ref.
Muslim	1.02 (0.81,1.23)	1.16 (0.92,1.46)	1.08 (0.87,1.35)
Sikh	1.03 (0.81,1.31)	1.08 (0.67,1.48)	1.07 (0.76,1.51)
Others	1.11 (0.87,1.43)	0.95 (0.69,1.31)	1.11 (0.82,1.49)
**Caste**			
Scheduled Caste	Ref.	Ref.	Ref.
Scheduled Tribe	0.82 (0.65,1.04)	0.84 (0.65,1.08)	0.81 (0.64,1.04)
Other Backward Class	1.07 (0.93,1.24)	0.79*(0.67,0.94)	0.96 (0.82,1.12)
Others	0.95 (0.83,1.09)	0.83*(0.71,0.98)	0.81*(0.7,0.95)
**Residence**			
Rural	Ref.	Ref.	Ref.
Urban	1.01 (0.91,1.12)	0.99 (0.87,1.12)	1.17*(1.04,1.32)
**State**			
Himachal Pradesh	Ref.	Ref.	Ref.
Punjab	2.06*(1.67,2.55)	0.38*(0.27,0.54)	0.72*(0.52,0.99)
West Bengal	3.87*(3.18,4.71)	1.92*(1.53,2.41)	4.88*(3.91,6.11)
Orissa	0.76*(0.63,0.92)	2.34*(1.86,2.95)	2.13*(1.69,2.68)
Maharashtra	0.73*(0.61,0.88)	1.63*(1.29,2.06)	3.31*(2.63,4.14)
Kerala	3.01*(2.48,3.66)	1.08 (0.82,1.41)	1.36*(1.05,1.77)
Tamil Nadu	1.23*(1.01,1.54)	5.07*(3.98,6.46)	2.77*(2.17,3.52)
R-square	0.152	0.178	0.187

*Ref* Reference, *if *p* < 0.05, *SRH* self-rated health, *ADL* Activities of Daily Living, *IADL* Instrumental Activities of Daily Living, *%* percentage, *AOR* Adjusted Odds Ratio

## Discussion

Maintaining good oral health is a key to present an older adult as a healthy person and improvise his/her longevity and wellbeing [36]. In the present study, tooth loss was encountered in 12.3% of the older participants. The low prevalence of tooth loss in low income countries may be related to low prevalence of dental caries due to consumption of non-refined carbohydrates [37].

The findings from the bivariate and multivariate analyses of tooth loss with measures of SRH, psychological health and subjective well-being as dependent variables were remarkably similar. Regardless of the direction of the association between oral status and health outcomes, analysis of our data indicates that poor oral health, poorer SRH, psychological health and subjective well-being co-exist among older adults. Similar finding pertaining to the present study found in the past literature indicating that the tooth loss in negatively associated with general health status and overall quality of life [37–40]. Loss of teeth also means loss of aesthetics in facial profile and personality, which does affect the social performance and ability of the individual to form social relations and low levels of well-being [17]. Further, it was also shown that the consequences of tooth loss that influence chewing ability, food intake and nutritional status, are complex leading to several problems in old age, including chronic diseases, functional disabilities, psychological and social factors, and lowered socioeconomic status [41, 42].

A review of literature reveals that poor health and disability are associated with low SWB at all ages and the health status is a strongest predictor of SWB during late life [43]. Consistently, our study also found that those who reported chronic diseases or low ADL or low IADL had poor SRH and low psychological and subjective well-being among the study sample. The performance of daily living activities and reduced functional dependency may strengthen feelings of autonomy and the ability to make personal decisions about how one should live in accordance with his/her own rules and preferences and that is fundamental to the mental health and well-being of older adults [44]. Moreover, the analysis also revealed a significant association between age and self-assessed health outcomes. This result was expected, considering the progressiveness of disability and dental caries among higher age groups, which account for many of the diseases.

Further, living with children and having greater number of children increase the likelihood of rotational living and shared responsibilities among adult children and caregiving for their older parents [45, 46]. With social and familial support, older individuals generally feel loved and become confident when dealing with health problems and have higher self-esteem and well-being [47]. Concordantly, the present study also shows that older adults who are living with spouse, children or other relatives reported better psychological and subjective well-being compared to solo living older adults. Whereas, study also found that quality of an individual’s relationships with spouse, children, and family rather than their presence was associated with psychological well-being [48].

Moreover, education and household economic status were found having significant negative relationship with poor SRH, low psychological health and low SWB. The finding is also consistent with previous studies showing that higher educational status and better economic conditions lead to higher levels of life satisfaction and well-being among older adults [49–51]. This is an interesting finding that indicates that lower-socio-economic group may not have either sufficient access or resources to seek dental treatment and leads to poor ratings of their health. In addition, educated older adults may have improved dental awareness, increased utilization of health facilities, proper oral hygiene habits that result in better oral health and well-being.

Studies of dental and oral problems among older individuals and their treatment seeking behaviour found that adequate access to medical and dental care can reduce premature morbidity and mortality, preserve function, and enhance overall quality of life [52, 53]. A study in India also found that less than 50% of edentulous and only 10% of partially edentulous older adults were wearing dentures due to the wide gap between the need and the services utilized [20].

The study has certain limitations. The response for tooth loss was self-reported in nature. Furthermore, there are chances of misreporting of information, as the information on SRH, psychological health and SWB was self-reported. Similarly, the study findings are also limited by the possible effects of residual confounding in the current analyses. Although the data presented here support contemporary thinking concerning tooth loss among older population and their well-being, the findings should be interpreted with caution. Since the study was predominantly cross-sectional, the direction of the causality remains unresolved. The data were collected from seven states only. However, the population from these seven states was representative of the national sample. Apart from the above limitations, the study has strengths too. The study provides the pan India prevalence for tooth loss among older adults which was a missing part form recent literature in India. Moreover, the study associated tooth loss with overall health among older adults which was a much needed area to be focused in the country like India where people associate tooth loss as a process of ageing and ignore the consequences that may worsen their overall health. Furthermore, longitudinal studies are warranted to broaden our current understanding of transitions in dental care during the late stages of life and its effect on well-being of older people.

## Conclusion

Tooth loss continues to represent a major health care burden that is often neglected in countries like India. Loss of tooth in later years is associated with older individuals’ poor SRH as well as low psychological and subjective well-being, but such a consequence is avoidable by practicing the efforts to maintain good oral health. Again, there is an increased need to understand the life context of the older individuals with less functional dentition in order to provide them with a better dental care through interventions capable of having a positive impact on the process of healthy ageing.

## Data Availability

The study utilises a secondary source of data that is freely available in public domain through request to http://www.isec.ac.in/.

## Abbreviations

BKPAI: Building Knowledge Base on Population Ageing in India
SRH: Self-rated health
GHQ: General Health Questionnaire
SWB: Subjective well-being
ADL: Activities of daily living
IADL: Instrumental activities of daily living
OR: Odds Ratio
CI: Confidence Interval
